# Refractory left pleural effusion in an older patient: Atypical presentation of colorectal carcinoma

**DOI:** 10.3892/ol.2015.2843

**Published:** 2015-01-05

**Authors:** YUJIE YUAN, JINNING YE, CHUANGQI CHEN, WU SONG, JIANBO XU, JIANHUI CHEN, YAPING XING, YULONG HE

**Affiliations:** Department of Gastrointestinal-Pancreatic Surgery, The First Affiliated Hospital of Sun Yat-Sen University, Guangzhou, Guangdong 510080, P.R. China

**Keywords:** pleural effusion, colorectal carcinoma, gastrointestinal stromal tumor, unusual case

## Abstract

The current study reports the case of a 60-year-old male that presented with a four-month history of recurrent chest pain. Chest X-ray examination revealed a left-sided pleural effusion. A closed thoracic drainage procedure was performed, but the chest pain relapsed shortly afterwards. The pleural fluid was exudative, with no tumor cells detected. A computed tomography scan subsequently revealed a large mass located in the splenic curve of the colon, with involvement of the greater curvature of the stomach. Endoscopic biopsies confirmed the diagnosis of adenocarcinoma. A gastrointestinal stromal tumor was excluded by endoscopic ultrasonography and biopsy. A subtotal colectomy with partial excision of the stomach, diaphragm and left liver lobe was successfully performed, followed by the administration of six cycles of adjuvant chemotherapy. At present, the patient is asymptomatic and there is no evidence of tumor recurrence following a 12-month follow-up. The present study summarizes the characteristics of refractory left pleural effusion by colorectal malignancies.

## Introduction

A number of primary colorectal carcinoma are often found by colposcopy or computed tomography (CT) scans. The detection of these tumors is mainly based on an active complaint of certain typical digestive symptoms, including abdominal pain, bright red blood in the stool and weight loss (1). However, tumors occasionally exhibit atypical presentations with a variety of symptoms or signs at a distance from the digestive system (2,3). Chest pain and pleural effusion are particularly uncommon presentations of digestive malignances that can be easily ignored in the Outpatient Department compared with other clinical presentations. A refractory pleural effusion presenting as a primary symptom of colorectal cancer is rarely reported (<0.9% incidence according to our own database) and mainly occurs in the elderly. In the present report, an older patient, who was admitted to The First Affiliated Hospital of Sun Yat-Sen University (Guangzhou, China) with a refractory left pleural effusion, was diagnosed with colon carcinoma and underwent a radical tumor resection. The clinical features of refractory pleural effusion that result from a colonic tumor are summarized, with a focus on the differential diagnosis of gastrointestinal stromal tumors. Written informed consent was obtained from the patient.

## Case report

**Figure 1:**
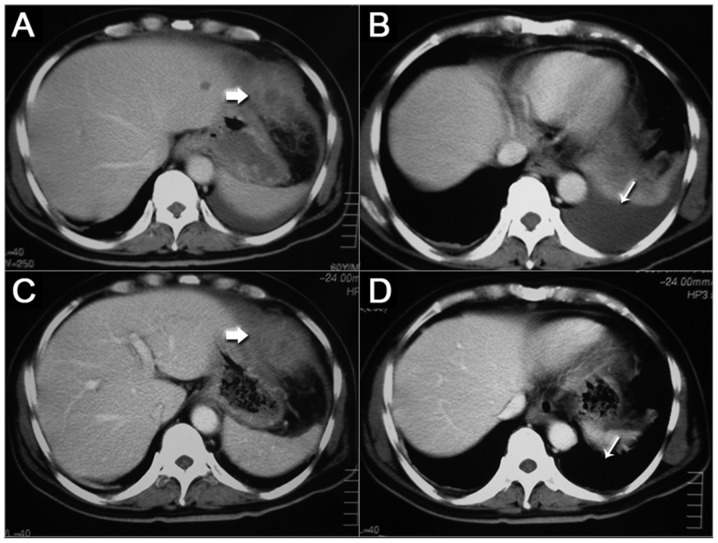
Computed tomography (CT) results of the patient prior to admission to The First Affiliated Hospital of Sun Yat-Sen University (Guangzhou, Guangdong, China). (A and B) Horizontal CT scan prior to a percutaneous thoracic drainage procedure. An extremely large mass, 20×15×8 cm in size, was located in the splenic curve of the colon, and a left-sided pleural effusion can be distinctly observed. (C and D) Horizontal CT scan following a percutaneous thoracic drainage procedure. Wide arrows indicate the mass under the diaphragm, whereas thin arrows mark the regions of pleural effusion. The resolved pleural effusion (D) reoccurred shortly after admission to The First Affiliated Hospital of Sun Yat-Sen University.

A 60-year-old male presented to the Outpatient Department of The First Affiliated Hospital of Sun Yat-Sen University due to a four-month history of repeated chest pain. The pain was located mainly on the left side of the chest and had worsened over the three days prior to admission. The patient had not developed a cough, excess sputum, hemoptysis or other symptoms. A chest X-ray examination performed at a local hospital revealed a left-sided pleural effusion. A closed thoracic drainage procedure was performed at this time, but the pain recurred in the same location shortly afterwards. The pleural fluid was exudative and was found to lack tumor cells by cytological analysis. A non-enhanced CT scan of the chest and abdomen revealed a marked soft-tissue prominence in the gastric greater curvature and a mass posterior to the spleen (Fig. 1). No intrahepatic lesions were found, and the other organs in the abdomen were unremarkable. The patient was referred to The First Affiliated Hospital of Sun Yat-Sen University (Guangzhou, China) for further diagnosis and treatment. Following admission, an ensuing examination was performed, consisting of a tumor marker examination for digestive malignancies, esophagogastroduodenoscopy, colonoscopy, additional staging by enhanced CT and endoscopic ultrasonography to identify the characteristics of non-presumed masses.

**Figure 2:**
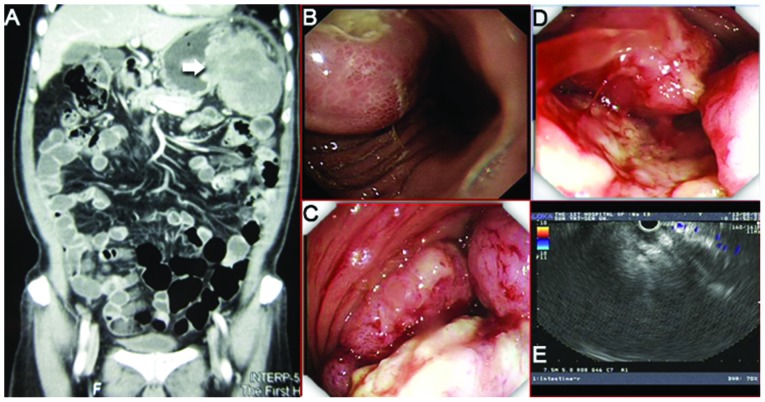
Radiographic findings of the patient following admission to The First Affiliated Hospital of Sun Yat-Sen University (Guangzhou, Guangdong, China). The mass was located under the diaphragm, with good demarcation and full invasion of all layers of the stomach and colon. (A) Coronary computed tomography scan. (B) Gastric endoscopy. (C and D) Colonoscopy. (E) Endoscopic ultrasonography. A total bowel obstruction was observed due to tumor invasion.

**Figure 3:**
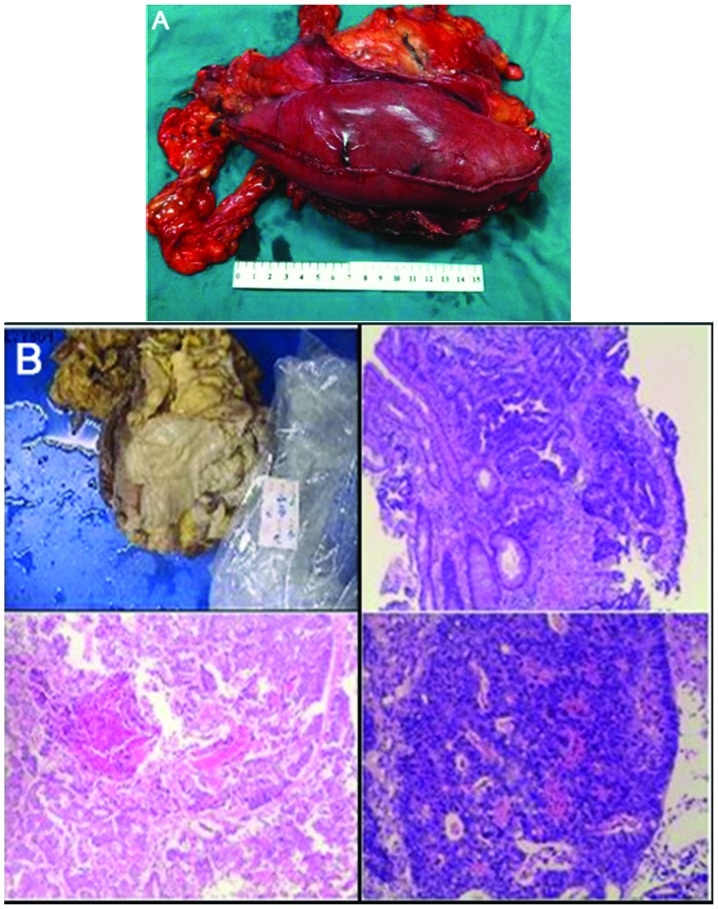
Final results following a definitive surgery. (A) Surgical resection of whole neoplasm. (B) Pathological presentation of the resected samples (hematoxylin and eosin staining; original magnification, ×100) indicating a primary moderately-differentiated colonic adenocarcinoma (mucinous type).

An increased carbohydrate antigen (CA)125 level (67.1 U/ml; normal range, 0–35 U/ml) was recorded, but the other markers were in the normal range. Endoscopic examination revealed a neoplasm, 6 cm in diameter, in the fundus of the stomach, along the greater curvature. Another cauliflower-like neoplasm was located at the splenic curve of the descending colon, which resulted in total bowel obstruction (Fig. 2). The biopsy results for the two masses indicated a diagnosis of a moderate- to poorly-differentiated adenocarcinoma. Taken together with findings from the endoscopic ultrasonography and abdominal enhanced CT (Fig. 2), the two neoplasms were confirmed to be homologous, without any observation of distal metastasis. Following a multiple-disciplinary team discussion for the present case, the clinical tumor-node-metastasis stage was set to cT4bN0M0, stage IIC, and a laparostomy was scheduled for a radical resection (R0). During the surgery, diaphragm and left hepatic lobe involvement was identified, and an extensive resection, which consisted of a partial greater curvature gastrectomy, segment 1 hepatic lobectomy, segmental left diagram resection, partial left abdominal wall resection and left hemicolectomy, was successfully performed (Fig. 3A).

Six days after the surgery, the patient was discharged from the department. The final diagnosis was moderately-differentiated colonic adenocarcinoma, with one out of 25 examined lymph nodes found to be positive for metastasis (pT4bN1aM0, stage IIIC; Fig. 3B). The patient was subsequently scheduled for approximately six courses of oxaliplatin 85 mg/m^2^ i.v. over 2 hours on day 1, leucovorin 400 mg/m^2^ i.v. over 2 hours on day 1 and fluorouracil 400 mg/m^2^ i.v. bolus on day 1, then 2400 mg/m^2^ over 48 hours by continuous i.v. infusion (mFOLFOX6; repeated every two weeks). Within the 12-month follow-up period, the patient was asymptomatic, without evidence of tumor recurrence.

## Discussion

The present study reported an unusual case of colorectal adenocarcinoma, with an atypical clinical presentation. The patient was admitted to hospital with non-specific symptoms of gastrointestinal malignancies, and a notable amount of time was spent on finding the etiology of the pleural effusion. Generally, refractory pleural effusion, which leads to the persistent complaint of chest pain, can be caused by various diseases, including bacterial pneumonia, lung cancer, heart failure and kidney dysfunction. Pleural effusion is often considered to be an intervention-requiring complication regardless of the primary disease. However, it is less common that the pleural effusion is raised by the chronic stimulation of intra-abdominal neoplasm. In that condition, ascites and peritoneal metastasis often develop prior to the pleural effusion in the majority of digestive or gynecological malignancies (4,5). However, in the present case, no ascites or peritoneal invasion was observed, with regional invasion analyzed by CT scan and endoscopic ultrasonography. For the diagnosis of a non-presumed pleural effusion, CT scans are particularly useful for detecting neoplastic disease of the upper abdomen prior to certain invasive procedures (6).

Among the various types of intra-abdominal neoplasms, digestive tumors, including intestinal lymphoma, gastrointestinal stromal tumors (GISTs) and colorectal cancer, are more frequently associated with pleural effusion compared with gynecological malignancies. In the present case, a GIST was also a possible diagnosis according to the features of the abdominal CT scan. Therefore, endoscopic ultrasonography was employed to discern between the two forms of digestive malignancy, as it is a useful tool for the accurate diagnosis of a GIST (7). Generally, a GIST is located in the muscularis propria, whereas colorectal cancer often invades all layers of the gastrointestinal wall (8). Uncommonly, intra-abdominal endometriosis can also induce similar symptoms to colon cancer and caution is therefore necessary when treating young females (9).

Pleural effusion is an atypical presentation of colorectal carcinoma, which exhibits its own features (3). According to our clincial experiences, this complication is always located in the left thoracic cavity and is frequently found in the elderly by a respiratory physician. Percutaneous thoracic drainage may be futile and inadequate to clear the effusion, with a sterile exudate obtained by pleural tap. Thoracic-abdominal CT scans identify a huge mass in the splenic curve of the colon, and endoscopic biopsies confirm the diagnosis of adenocarcinoma. At present, there are no studies to indicate that GIST is relevant to this specific complication. In the majority of cases, the diaphragm, stomach, left liver lobe and partial peritoneum are vulnerable and require excision during a subtotal colectomy procedure, with several courses of subsequent adjuvant chemotherapy. If a radical resection is achieved and the subsequent chemotherapy is successfully completed, the pleural effusion is quickly resolved and the prognosis of colonic adenocarcinoma is quite good.

Older patients with left pleural effusion, but no presumed diagnosis arising from standard clinical examination, as in the present case, should undergo thoracic-abdominal CT scans to exclude potential upper abdominal neoplasms. Endoscopic ultrasonography is useful for the exclusion of GISTs and to guide the subsequent surgical treatment. A radical tumor resection combined with adjuvant chemotherapy is indispensable for colorectal adenocarcinoma elimination, and the prognosis of this specific cancer is quite favorable.

In conclusion, the successful management of the present case indicates that refractory pleural effusion is an atypical presentation of colorectal cancer. Early diagnosis and radical resection would improve the long-term outcomes of colorectal cancer patients.
